# Microwave Differential Frequency Splitting Sensor Using Magnetic-LC Resonators

**DOI:** 10.3390/s20041066

**Published:** 2020-02-15

**Authors:** Amir Ebrahimi, Grzegorz Beziuk, James Scott, Kamran Ghorbani

**Affiliations:** School of Engineering, RMIT University, Melbourne, VIC 3001, Australia; greg.beziuk@rmit.edu.au (G.B.); james.scott@rmit.edu.au (J.S.); kamran.ghorbani@rmit.edu.au (K.G.)

**Keywords:** differential sensors, microwave sensors, microwave comparator, permittivity sensing

## Abstract

A differential microwave permittivity sensor and comparator is designed using a microstrip transmission line loaded with a magnetic-LC resonator. The microstrip transmission line is aligned with the electric wall of the resonator. The sensor shows a single transmission zero, when it is unloaded or loaded symmetrically on both halves. A second notch appears in the transmission response by asymmetrical dielectric loading on the two halves of the device. The frequency splitting is used to characterize the dielectric properties of the samples under test. The sensitivity of the sensor is enhanced by removing the mutual coupling between the two halves of the magnetic-LC resonator using a metallic wall. The sensors’ operation principle is explained through a circuit model analysis. A prototype of the designed sensor is fabricated and measurements are used for validation of the sensing concept. The sensor can be used for determination of the dielectric properties in solid materials or detecting defects and impurities in solid materials through a comparative measurement with a reference sample.

## 1. Introduction

Metamaterials-based components, such as split-ring resonators (SRRs) and complementary split-ring resonators (CSRRs) have been widely used in the implementation of high performance compact microwave electronic components such as filters and antennas [1,2,3,4,5,6]. In recent years, metamaterials-based resonators have found numerous applications in sensing and measurements. RF and microwave sensors are popular in various measurement and instrumentation applications due to their robust, real-time, and label free measurement compatibilities [7,8,9,10,11,12,13,14,15,16,17,18,19,20]. The measurement principle in a majority of microwave sensors is the frequency shift or notch magnitude variation, where any environmental or structural alterations leads to modification of the resonance characteristics [21,22,23,24,25,26,27,28,29,30,31]. Like any other types of sensing or measurement tools, the measurement accuracy of the microwave-based sensors is affected by the cross-sensitivity (a similar reaction of the sensor to a parameter rather than the parameter of interest) to the environmental factors such as temperature, humidity, etc. Recently, a new class of microwave sensors have been developed with inherent robustness with respect to the cross-sensitivity. This class of sensors are called differential sensors [32,33,34,35,36,37,38,39,40,41,42,43,44]. In the differential sensors, variations in the ambient factors are seen as a common mode input. Therefore, their effect is suppressed by the differential detection mechanism in the sensor.

The differential permittivity sensors are usually implemented using two symmetrical sensing elements, where any asymmetrical loading of the sensing elements causes frequency splitting or change in the cross-mode *S*-parameters of the sensor. The differential sensors using the cross-mode *S*-parameters presented in [32,33,34] show a very high sensitivity to the variations in the loss tangents of the samples under test, as the cross-mode *S*-parameters levels are very sensitive to the loss tangent of the samples. Generally, the frequency splitting differential sensors in [35,36,37,38,39] are more appropriate for low loss dielectrics as the resonance frequency shows a high sensitivity to the variations in the real part of the permittivity. The differential sensors in [32,33,34] are four port devices thereby requiring a more complicated measurement setup. Split-ring resonators (SRRs) are used for designing differential permittivity sensors in [35]. Generally, the capacitive effect and as a result the fringing electric field provide by SRRs is smaller than the complementary counterparts such as CSRRs and magnetic-LC (MLCs) (complementary electric-LC (ELC)) [45]. This causes a smaller sensitivity of the SRR-based sensors to the dielectric variations with respect to the complementary resonators-based sensors. The differential sensor in [36] is implemented with a microstrip transmission line loaded with two step impedance resonators (SIRs). A half-wavelength distance between the two sensing elements is required in the stepped impedance resonator-based sensor in [36] causing a large device area, which is not appropriate for integrated platforms. Accurate design of the splitter/combiner feedlines are required to achieve optimum sensitivity in [37,38]. In addition, a large spacing is required between the sensing elements in [39] to avoid coupling and sensing degradation.

Here, we present a frequency splitting differential sensor using a microstrip transmission line loaded with a MLC resonator. The complementary nature of the MLC causes a high electric field concentration at resonance. This results in a high sensitivity of the sensor to any dielectric loadings. The designed sensor is a two port device requiring a simpler measurement in network analyzer test setups. Furthermore, the inherently small nature of the MLC resonator offers a compact sensor size appropriate for integrated systems. The electric wall (a plane, where the electric fields are distributed symmetrically on its’ two sides) of the MLC is aligned with the horizontal symmetry line of the microstrip transmission line. This leads to a balanced excitation of the two resonator halves causing a single notch in the transmission response of the bare sensor. However, any unbalanced loading causes asymmetry in the resonator and excites the odd resonance mode causing a second transmission notch and frequency splitting. This characteristic is used for differential characterization of the unknown test samples.

## 2. Description of the Sensor Structure and Operation Principle

**Figure 1:**
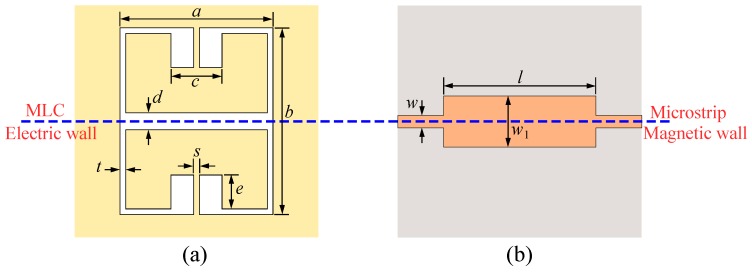
Layout of a microstrip transmission line loaded with a magnetic-LC resonator. (**a**) Bottom view and (**b**) top view. The microstrip metallization is shown in orange, the ground plane metallization in yellow, and the dielectric substrate is shown with gray color. The geommetrical dimensions are a=10.2 mm, b=15.4 mm, c=2.6, d=2.2 mm, e=2.6 mm, s=t=0.2 mm, w=1.2 mm, and $w_{1}=6.2$ mm.

**Figure 2:**
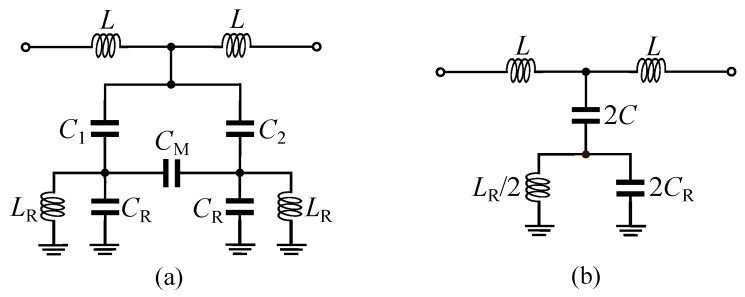
Equivalent circuit model of a microstrip line loaded with a MLC resonator. (**a**) General circuit model of the structure. (**b**) The circuit model, when the MLC electric wall is aligned with the magnetic wall of the microstrip transmission line.

The proposed sensor is designed based on a microstrip transmission line loaded with a MLC resonator as shown in Figure 1. The electric wall of the MLC resonator is aligned with the magnetic wall (a plane, where the magnetic fields are distributed symmetrically on its two sides) of the microstrip transmission line. Such a structure is analyzed in detail in [46]. A general equivalent circuit model of the structure is presented in Figure 2a. In the circuit model, the $L_{R}C_{R}$ and $C_{M}$ model the MLC resonator, where $C_{R}$ models the capacitive effect between each rectangular half of the resonator and the surrounding ground plane and $L_{R}$ is the inductance of the thin metallic traces connecting the rectangular halves to the ground plane. The $C_{M}$ is the mutual capacitance between the two halves of the MLC resonator. The *L* inductances model the equivalent inductance of the short microstrip line sections above the MLC resonator. Finally, $C_{1}$ and $C_{2}$ model the capacitive couplings between the microstrip transmission line and the two sides of the MLC resonator. When the microstrip transmission line is aligned with the resonator, as shown in Figure 1, the $C_{1}$ and $C_{2}$ capacitors will take the same value ($C_{1}=C_{2}=C$). Therefore, there would be no voltage difference across the $C_{M}$ capacitor and the circuit takes the form shown in Figure 2b. In this condition, the transmission response ($S_{21}$) of the structure shows a single bandstop notch at $f_{z}$ defined as follows,

(1) $$f_{z}=\frac{1}{2\pi \sqrt{L_{R}\left(C+C_{R}\right)}}.$$

**Figure 3:**
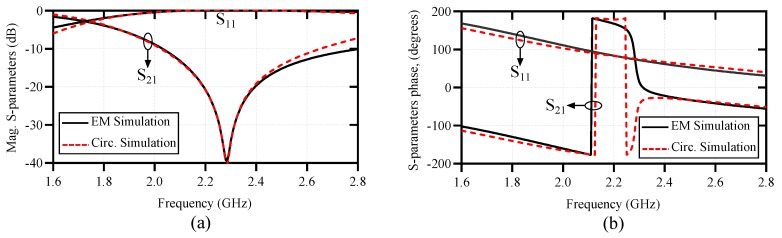
Comparison between the full-wave electromagnetic and the circuit model simulation results of a microstrip line loaded with MLC resonator, when the electric wall of the resonator is aligned with the magnetic wall of the microstrip transmission line. (**a**) Amplitude response and (**b**) phase response. The extracted lumped element values are L=4.7 nH, C=1.162 pF, $C_{R}=1.94$ pF, and $L_{R}=1.566$ nH.

To validate the above analysis, a comparison between the equivalent circuit model and the full-wave electromagnetic (EM) simulation results of the structure in Figure 1 is presented in Figure 3. The geometrical dimensions of the structure are given in the caption of Figure 1, and the substrate used for the simulations is a 0.508 mm thick Rogers RT6002 with $\varepsilon_{r}=2.93$ and a loss tangent of 0.0013. The agreement between the results in Figure 3 verifies the above analysis. The difference between the EM and circuit model results is mostly attributed to the mismatch between the 50Ω short input sections and the wide microstrip line section above the resonator part. The circuit does not model the transition between the 50Ω feeds and the wide microstrip line section. Now, if the symmetry is broken, a voltage difference will appear across the $C_{M}$ capacitor. As explained in [46], this excites the odd-mode resonance of the MLC causing a second bandstop notch in the transmission response of the structure. The symmetry might be broken in two ways: (i) by displacing the microstrip transmission line with respect to the electric wall of the MLC and (ii) by asymmetrically loading the halves of the MLC resonator with dielectric samples. By displacing the microstrip transmission line with respect to the MLC’s electric wall, the $C_{1}$ and $C_{2}$ capacitors take unequal values causing an asymmetry in the circuit. On the other hand, for asymmetrical dielectric loading on the two halves of the MLC, the two halves of the resonator show an unbalanced capacitive effect ($C_{R}$ would be unequal for the two halves) causing asymmetry in the circuit.

**Figure 4:**
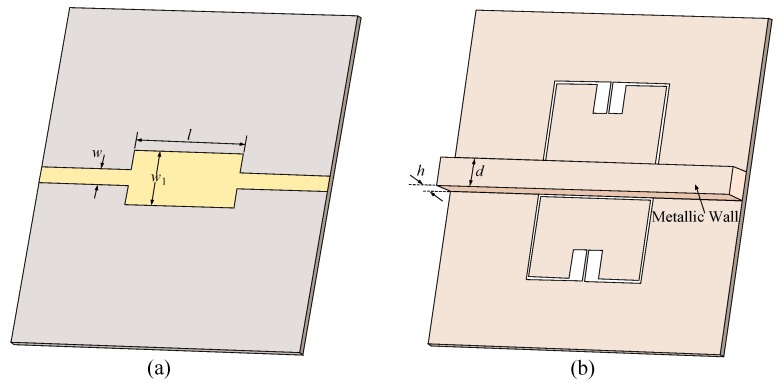
The designed differential sensor using a metallic wall separating the two halves of a MLC resonator. (**a**) Front view of the sensor. (**b**) Back view of the sensor. All the dimensions are the same as Figure 1 except $w_{1}=4.2$ mm and e=2.2 mm here. In addition, d=2.2 mm and h=2 mm.

**Figure 5:**
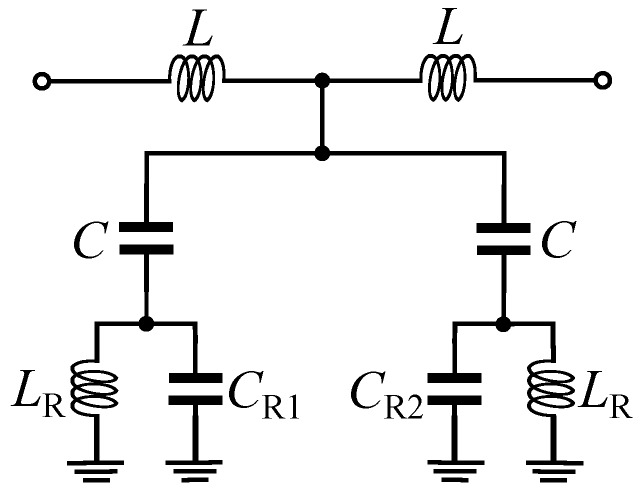
Equivalent circuit model of the sensor in Figure 4.

The basic operation principle of the differential sensor here is to introduce asymmetry by loading different dielectric materials into the two halves of the sensor. As explained above, this causes unbalanced $C_{R}$ values on the two sides of the resonator producing a second zero in the transmission response of the device. The relative permittivity of the sample under test can be obtained using the frequency difference between the two transmission zeros. However, the existence of capacitive coupling ($C_{M}$) degrades the sensitivity of the differential sensor [35,36,39]. In fact, by having a coupling between the two sensing elements (two halves of the resonator), any dielectric loading on one half of the sensor shifts both of the zero frequencies down. This reduces the frequency difference between them causing less sensitivity of the difference frequency with respect to the permittivity of the sample under test. To remove the capacitive coupling, a metallic wall is placed between the two halves of the MLC resonator as shown in Figure 4. In this configuration, $C_{M}$ is removed from the equivalent circuit model of the sensor and the circuit takes the form shown in Figure 5. The metallic wall must be high enough to prevent any electric field going from one side of the resonator to the other side. This height is optimized using simulations to completely decouple the two sides of the resonator. If there is any coupling between the two sides, by loading a dielectric slab on one side of the resonator frequency splitting happens, but the higher resonance frequency would also change instead of remaining constant. The *d* thickness should be larger than the skin depth at the operational frequency of the sensor. In reality, *d* is limited by fabrication, as it is challenging to fabricate a very thin wall. If the sensor is unloaded, then $C_{R1}=C_{R2}=C_{R}$ and the equivalent circuit model takes the form in Figure 2b. In this condition, there would be just one zero in the transmission response of the sensor. The frequency of this transmission zero is calculated as (1). Note that for the bare sensor in Figure 4, $C_{R1}$ and $C_{R2}$ are both larger than $C_{R}$ in the configuration of Figure 1. This is due to an additional capacitive effect between the metallic wall (that is attached to the ground plane) and the two rectangular patches of the MLC resonator. Now, if one of the sensing channels (resonator halves on the side of the metallic wall) is loaded with a test dielectric sample, then $C_{R1}\neq C_{R2}$ and two zeros appear in the transmission response of the sensor. The frequency of these transmission zeros are obtained as follows,

(2) $$f_{z}1=\frac{1}{2\pi \sqrt{L_{R}\left(C+C_{R1}\right)}},$$

(3) $$f_{z}1=\frac{1}{2\pi \sqrt{L_{R}\left(C+C_{R2}\right)}}.$$

**Figure 6:**
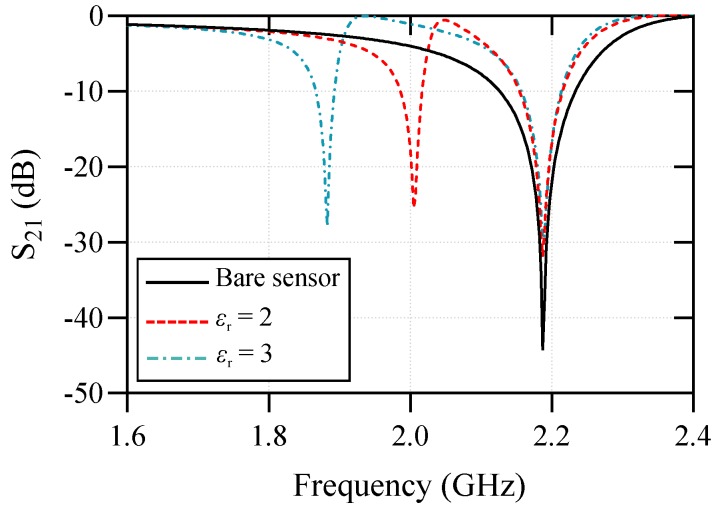
Simulated $S_{21}$ for the bare sensor and when one of the sensing elements is loaded with a test sample of $\varepsilon_{r}=2$ and $\varepsilon_{r}=3$.

Here, the sensing variable is the frequency difference ($f_{d}=f_{z1}-f_{z2}$), where one sensing channel is always unloaded and considered as a reference. This means $f_{z1}$ is always constant, whereas $f_{z2}$ is a function of the dielectric constant of the sample under test. A higher permittivity of the sample under test results in lower $f_{z2}$ and higher $f_{d}$. This is shown through the full-wave simulation results of the sensor, where the test channel is loaded with dielectric slabs of $\varepsilon_{r}=2$ and $\varepsilon_{r}=3$ in Figure 6. Here, by the relative permittivity or dielectric constant we mean the real part of the complex relative permittivity, and the focus of the presented work is permittivity measurement in low loss solid materials, where the imaginary part of the permittivity is much smaller than the real part.

## 3. Experimental Results

**Figure 7:**
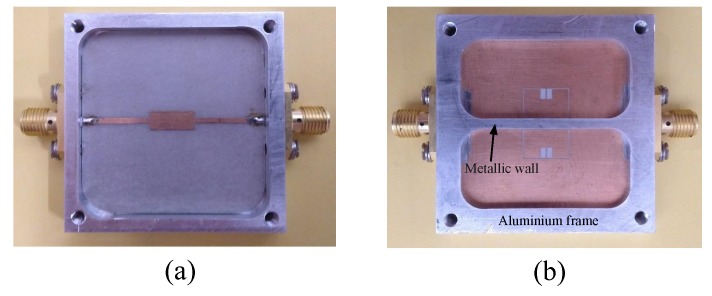
The fabricated sensor prototype (**a**) front view and (**b**) back view.

A prototype of the sensor is fabricated and tested for verifying the proposed differential frequency splitting sensing principle. The substrate used for fabrication is a 0.508 mm thick Rogers RT6002 with a relative permittivity of 2.93 and a loss tangent of 0.0037. The front and back views of the fabricated sensor prototype are presented in Figure 7. An aluminum frame is designed and attached to the sensor keeping it from bending and potential damages during the measurements. The metallic wall separating the two sides of the sensor is machined out of the aluminum frame.

**Figure 8:**
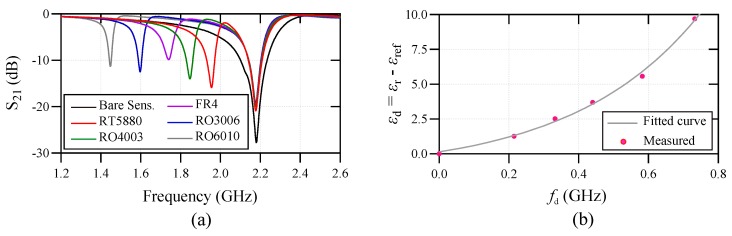
(**a**) The measured transmission responses of the sensor for various test dielectric samples. (**b**) The measured and curve fitted frequency splitting for various dielectric samples.

The sensor has been tested with five low loss dielectric slabs. The test dielectric slabs are 1.57 mm thick Rogers RT5880 with $\varepsilon_{r}=2.2$ and tanδ=0.0004, 1.524 mm thick Rogers RO4003 with $\varepsilon_{r}=3.55$ and tanδ=0.0027, 1.5 mm thick FR4 with $\varepsilon_{r}=4.7$ and tanδ=0.025, 1.28 mm thick Rogers RO3006 with $\varepsilon_{r}=6.5$ and tanδ=0.002, and 1.27 mm thick Rogers RO6010 with $\varepsilon_{r}=10.7$ and tanδ=0.0023. The samples were cut in $15\times 20\;{\mathrm{mm}}^{2}$ covering the whole sensing area. The dielectric samples were all thicker than 1.1 mm to remove the cross-sensitivity with respect to the thickness. The dielectric samples should be large enough to cover the whole resonator area on each side. The samples are simply placed on the top of the resonator with no applied pressure for keeping the consistency during the measurements. The measured transmission coefficients of the sensor for these samples are plotted versus frequency in Figure 8a. In all measurements, the reference sensing channel is unloaded (reference is the bare side of the sensor). Based on Figure 8a, the higher resonance frequency, which is attributed to the reference channel, is always constant at 2.18 GHz resembling the unloaded condition. Based on the measured results in Figure 8a, a mathematical sensing model is developed using the curve-fitting in MATLAB for determining the relative permittivity of the samples under test, using the difference between the resonance frequency of the reference sample and the sample under test. The obtained mathematical sensing model is as follows,

(4) $$\Delta \varepsilon_{r}=0.11527-1.47\left(1-\exp\left(2.738f_{d}\right)\right),$$

where, $\Delta \varepsilon_{r}=\varepsilon_{r}-\varepsilon_{\mathrm{ref}}$ is the difference between the permittivity of the sample under test and the reference sample, and $f_{d}$ is the difference between the resonance frequencies of the test and reference sensing channels, where the reference channel is unloaded meaning $\varepsilon_{\mathrm{ref}}=1$. The relative permittivity of each test sample is then calculated using (4) and the results are tabulated in Table 1. As seen, the maximum permittivity measurement error using the designed sensor is less than 5.2%, showing a high accuracy of the proposed sensor in determination of the relative permittivity of unknown dielectric samples. The actual $\varepsilon_{r}$ values in Table 1 are taken from the commercial datasheets provided by Rogers Corporation [47].

**Figure 9:**
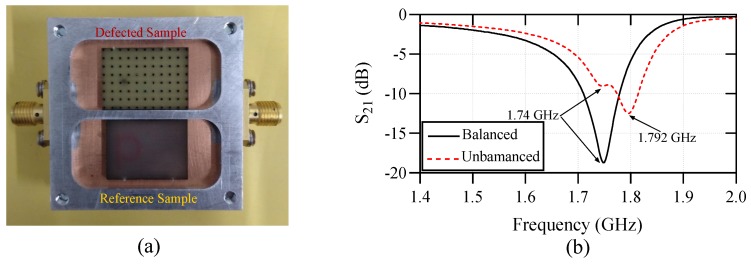
(**a**) The sensor setup as a comparator using a perfect and a defected FR4 dielectric slabs. (**b**) The measured responses of the sensor with balanced and unbalanced loadings of FR4 samples.

To investigate the functionality of the sensor as a comparator, it has been loaded with a perfect FR4 dielectric slab on the reference channel and a defected FR4 slab on the test sensing channel as shown in Figure 9a. The defects are implemented by laser milling of a hole array with each hole having a diameter of 1 mm and the center to center spacing of the adjacent holes is 2 mm. The measured transmission response of the sensor for this unbalanced loading is plotted in Figure 9b, where the transmission response for the balanced case (both channels loaded with perfect FR4 sample) is plotted as well. As seen in the plot, frequency splitting happens for the unbalanced loading, where the lower resonance frequency is exactly the same as the balanced case and the higher resonance frequency is happening at 1.792 GHz. As the perfect and defected FR4 samples have relatively close permittivity values, the two resonances are close to each other forming weaker stopbands compared to the balanced case. Based on the sensing model in (4), the defects decrease the effective permittivity of the FR4 dielectric to 3.9.

Table 2 presents a comparison between the proposed differential sensor and the state-of-art frequency splitting sensors in the literature in terms of various performance metrics. In the table, $f_{0}$ presents the resonance frequency of the unloaded sensor. The average sensitivity ($S_{\mathrm{Av}.}$) of each sensor is presented in the table. The average sensitivity is defined as

(5) $$S_{\mathrm{Av}}.=\frac{1}{n}\sum_{k=1}^{n}\frac{f_{d,k}}{\left(\varepsilon_{r,k}-1\right)},$$

where *n* is the number of test samples (5 here), $f_{d,k}$ is the frequency splitting of the *k*th sample, and $\varepsilon_{r,k}$ is the dielectric constant of the *k*th sample. As seen, the operation frequencies of the sensors in the literature are very different from each other. Therefore, for a fair comparison, the normalized sensitivity ($S_{\mathrm{Av}.,f}$) is presented, where ($S_{\mathrm{Av}.,f}$) is the average sensitivity normalized to $f_{0}$ for each sensor. Based on the table, the proposed sensor offers a very competitive performance compared to the rest of the designs in the literature. The designed sensor has the smallest size among the others offering more compatibility for integrated measurement systems. In addition, it shows a higher sensitivity with respect to the others except the sensor in [36]. Note that the sensing element in [36] is a cavity below the capacitive patch of the SIR, forming a parallel capacitance with the ground plane. This causes a higher fringing electric field for sensing. However, this sensor has a larger size with respect to the rest of the designs because of the $\lambda_{g}/2$ ($\lambda_{g}$ is the guided wavelength at the resonance frequency of the bare sensor) spacing between the two sensing channels to avoid coupling between them.

## 4. Conclusions

A frequency splitting differential permittivity sensor is designed using a microstrip transmission line loaded with a magnetic-LC (MLC) resonator. A complementary structure of the resonator increases the fringing electric field, which results in a high sensitivity of the proposed sensor to the dielectric loadings. The two sensing channels of the sensor are decoupled using a metallic wall improving the differential sensing performance. The design concept has been verified by fabricating a sensor prototype and testing it with various dielectric test samples. A mathematical sensing model has been developed for measuring the relative permittivity of unknown samples based on the frequency splitting principle. The measurements and comparisons with the state-of-art sensors in the literature prove the high performance of the designed sensor in determining the relative permittivity of unknown solid dielectric materials.

## Figures and Tables

**Table 1:** Comparison between the measured permittivities of the samples under test and using (4) and the actual values taken from the commercial datasheets.

Sample	Meas. $f_{d}$ (GHz)	Calc. $\varepsilon_{d}$	Meas. $\varepsilon_{r}$	Actual $\varepsilon_{r}$	Error (%)
RT5880	0.21	1.258	2.258	2.2	2.6
RO4350	0.347	2.44	3.44	3.55	3
FR4	0.44	3.55	4.55	4.7	3.2
RO3006	0.58	5.84	6.84	6.5	5.2
RO6010	0.7325	9.57	10.57	10.7	1.2

**Table 2:** Comparison between the performance of the designed frequency splitting sensor and the state-of-art ones.

Ref.	$f_{0}$ (GHz)	$S_{\mathrm{Av}.}$ (MHz)	$S_{\mathrm{Av}.,f}$(%)	Size	Differential	Planar
[35]	2.1	72	3.4	$0.034\lambda_{g}^{2}$	Yes	Yes
[36]	6.1	0.536	8.8	$0.15\lambda_{g}^{2}$	Yes	Yes
[37]	3	54.3	1.81	$0.064\lambda_{g}^{2}$	Yes	Yes
[38]	1.7	33.3	1.96	$0.098\lambda_{g}^{2}$	Yes	Yes
[48]	0.87	0.79	0.91	$0.1\lambda_{g}^{2}$	Yes	Yes
This Work	2.18	88	4.03	$0.02\lambda_{g}^{2}$	Yes	No
